# Supplementary material for: Tailoring supercurrent confinement in graphene bilayer weak links
Source: Nat Commun. 2018 Apr 30;9:1722. doi: 10.1038/s41467-018-04153-4 (PMC5928064; doi:10.1038/s41467-018-04153-4)
Supplement: Supplementary file 1 — Supplementary Information [file 41467_2018_4153_MOESM1_ESM.pdf]

Supplementary Informations for
Tailoring supercurrent confinement in graphene bilayer weak links

Rainer Kraft,[1] Jens Mohrmann,[1] Renjun Du,[1] Pranauv Balaji Selvasundaram,[1,2] Muhammad Irfan,[3,4] Umut Nefta Kanilmaz,[1,5] Fan Wu,[1,6] Detlef Beckmann,[1] Hilbert von Löhneysen,[1,7,8] Ralph Krupke,[1,2] Anton Akhmerov,[3] Igor Gornyi,[1,5,9] and Romain Danneau[1]

[1]*Institute of Nanotechnology, Karlsruhe Institute of Technology, D-76021 Karlsruhe, Germany*

[2]*Department of Materials and Earth Sciences, Technical University Darmstadt, Darmstadt, Germany*

[3]*Kavli Institute of Nanoscience, Delft University of Technology, P.O. Box 4056, 2600 GA Delft, The Netherlands*

[4]*Department of Physics and Applied Mathematics, Pakistan Institute of Engineering and Applied Sciences, Nilore, Islamabad 45650, Pakistan*

[5]*Institute for Condensed Matter Theory, Karlsruhe Institute of Technology, D-76128 Karlsruhe, Germany*

[6]*College of Optoelectronic Science and Engineering, National University of Defense Technology, Changsha 410073, China*

[7]*Institute of Physics, Karlsruhe Institute of Technology, D-76049 Karlsruhe, Germany*

[8]*Institute for Solid State Physics, Karlsruhe Institute of Technology, D-76021 Karlsruhe, Germany*

[9]*A.F. Ioffe Physico-Technical Institute, 194021 St. Petersburg, Russia*

SUPPLEMENTARY NOTE 1: DEVICE FABRICATION

The edge connected van der Waals heterostructures based on hBN-bilayer graphene-hBN are prepared following the method developed by Wang *et al.* [1]. Bilayer graphene (BLG) flakes and hexagonal boron nitride multilayers (bottom and top hBN multilayer are ~ 35 nm and ~ 38 nm thick respectively) are obtained by mechanical exfoliation from natural bulk graphite (NGS Naturgraphit GmbH) and commercial hBN powder (Momentive, grade PT110) respectively and transferred on *p*-doped Si substrates with 300 nm thick thermally grown SiO_2 layer and selected by optical contrast [2]. Raman spectroscopy was used to unambiguously identify BLG [3] (Renishaw inVia Raman spectrometer, using a laser of wave length $\lambda = 532$ nm). The graphene was then encapsulated between a top and a bottom hBN flake by piling up the layers sequentially by using a polymer-free assembly technique [1] and a home-made transfer set-up. Then, the whole stack was transferred onto a sapphire substrate with a Cr/Au (5 nm/50 nm) pre-patterned back-gate which is covered by a dielectric of 20 nm Al_2O_3 deposited by atomic layer deposition [4] (182 cycles at 200 °C). Edge contacts are designed on the mesa and defined by electron beam lithography using a single resist layer of PMMA covered by conductive polymers (Espacer 300Z from Showa Denko K.K., see [5]) for both etching and subsequent metallisation. The conductive polymer insures good evacuation of charges allowing e-beam lithography on a fully insulating substrate such as sapphire. Unlike in [1], only PMMA was used as a mask (~ 250 nm). The hBN-BLG-hBN sandwich was then etched in an Oxford Instruments Plasmalab 80 reactor with a mixture of CHF_3 and O_2 forming a 60 W plasma (40 sccm CHF_3 with 4 sccm O_2 at a pressure of 60 mTorr; etching rate: 23 and 48 nm/min for PMMA and hBN respectively). A double layer of titanium/aluminium (5 nm/80 nm) electrodes were then deposited by molecular beam epitaxy (at pressure and sample temperature ~ 10^{-10} mTorr and ~ −130 °C respectively) using the same already patterned PMMA resist, followed by lift-off in acetone. In a subsequent step, split-gates are fabricated in the similar fashion, *i.e.* e-beam lithography followed by metallisation (a Ti/Al double layer of 5 nm and 80 nm thickness respectively). It is important to note that the width of the split-gates (~ 300 nm) does not exceed two times the London penetration depth ($\lambda_L > 200$ nm in aluminium thin films according to [6]) so that the applied magnetic field is not disturbed by the split-gate electrodes becoming superconducting at very low temperature. Finally, the devices are shaped into the desired geometry by a third lithography step and subsequent etching using the parameters of the previous etch process. The distance between the two fingers of the split-gate is $w \sim 65$ nm.

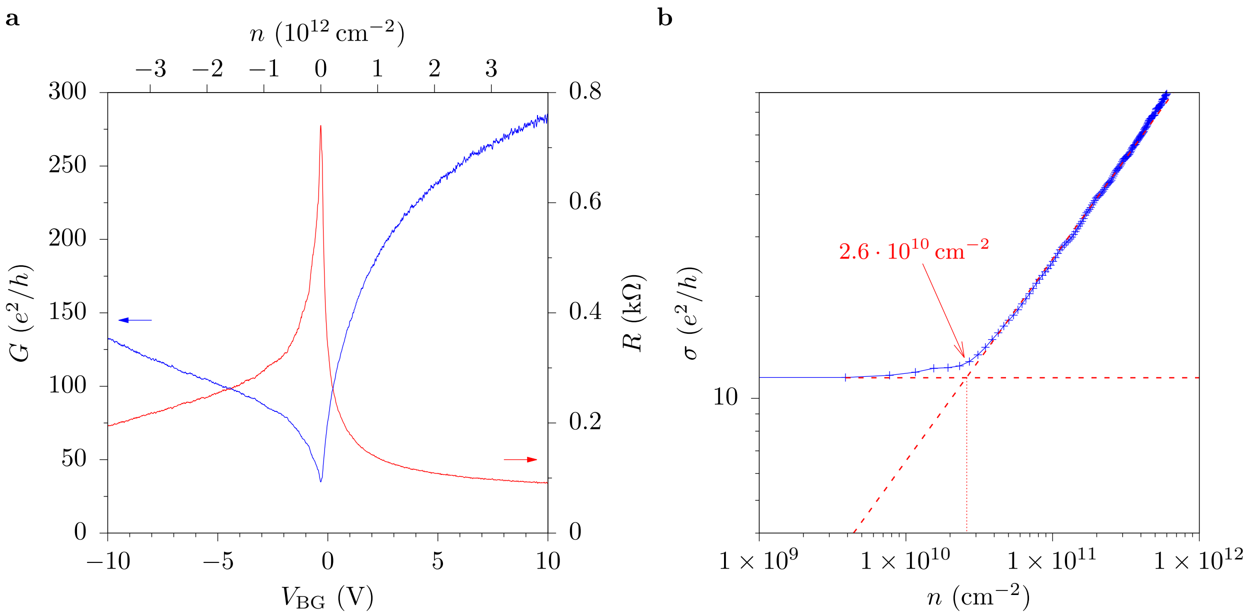

SUPPLEMENTARY FIGURE 1. **Normal state characterisation. a,** Resistance R (red curve) and conductance G (blue curve) as a function of the back-gate voltage V_{BG} (lower abscissa) or converted charge carrier density n (upper abscissa) of the 2D system respectively. The curve was measured in a separate cooldown at a split-gate voltage $V_{SG} = V_{SG}^{(0)} = 0.2\,$V. **b,** log-log-plot of the conductivity σ as a function of n. A contact resistance of $2R_C = 72\,\Omega$ was subtracted. The crossing of the saturation conductivity with the extrapolated linear fit to the data at high charge carrier density yields a residual charge inhomogeneity of the order of $10^{10}\,$cm^{-2}. It is important to note that clear Fabry-Pérot resonances (see Fig. 2 of the main text and Supplementary Figure 3) are visible in all cavities formed by the split-gate or by the unintentional doping from the leads, indicating ballistic transport across the device.

SUPPLEMENTARY NOTE 2: NORMAL STATE CHARACTERIZATION

Supplementary Figure 1a shows both normal state resistance R and conductance G as a function of back-gate voltage V_{BG} and charge carrier density n, while Supplementary Figure 1b displays the electron conductivity (minus the estimated contact resistance calculated below) *vs* charge carrier density.

The contact resistance per contact is estimated as $R_C = (R - R_Q)/2$, where the quantum resistance R_Q is subtracted from the measured resistance R. The quantum resistance $R_Q = \frac{h}{ge^2}\frac{1}{M}$ is defined as the resistance set by the ballistic limit of all contributing conductance modes $M = \frac{W}{\lambda_F/2}$, where $\lambda_F = 2\pi/k_F = 2\pi/\sqrt{\pi n}$ is the Fermi wavelength at charge carrier density n and $g = 4$

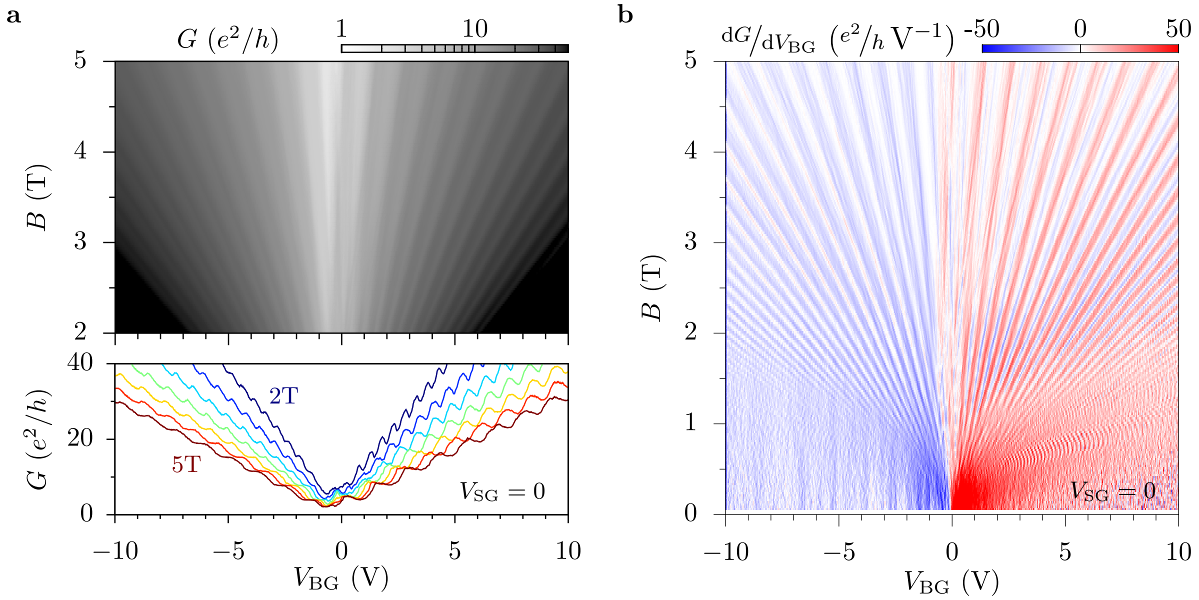

SUPPLEMENTARY FIGURE 2. **Two-terminal magnetotransport of the 2D bilayer graphene.** Landau fan diagram of, **a,** the two-terminal conductance $G(B, V_{\mathrm{BG}})$ and **b,** the two-terminal differentiated conductance $\mathrm{d}G/\mathrm{d}V_{\mathrm{BG}}(B, V_{\mathrm{BG}})$ as a function of the magnetic field B and the back-gate voltage V_{BG}, measured at a temperature ~ 25 mK.

accounts for the spin and valley degeneracy. At high charge carrier density $n \approx 4 \cdot 10^{12}\,\mathrm{cm}^{-2}$ ($M = 361$ and $R_{\mathrm{Q}} = 18\,\Omega$) a resistance of $R = 90\,\Omega$ is measured, yielding the contact resistance $R_{\mathrm{C}} = 36\,\Omega$ and contact resistivity $\rho_{\mathrm{C}} = R_{\mathrm{C}}W = 115\,\Omega\mu\mathrm{m}$, comparable to the values given by Wang *et al.* [1].

We estimate the residual density of $n_{\mathrm{res}} \approx 2.6 \cdot 10^{10}\,\mathrm{cm}^{-2}$ as to [7] on the electron side (see Supplementary Figure 1a). Supplementary Figure 2a displays a grey-scale map of the two-terminal conductance $G(B, V_{\mathrm{BG}})$ (upper panel) and $G(V_{\mathrm{BG}})$ curves for various B (lower panel). The two-terminal differentiated conductance $\mathrm{d}G/\mathrm{d}V_{\mathrm{BG}}$ map as a function of the magnetic field B and the back-gate voltage V_{BG} is shown in Supplementary Figure 2b. The two-terminal quantum Hall conductance curves display distorted conductance plateaus as expected for high aspect ratio W/L sample geometry [8–12]. One can observe a well developed Landau fan even at relatively low magnetic field, highlighting the high quality of our device.

We provide quantitative estimation of the gap induced by the displacement field that break the lattice symmetry underneath the split-gate using the formula provided in [13, 14]. The gap should be larger than the potential fluctuation coming from the residual charge carrier inhomogeneity

$n_{res} \approx 2.6 \cdot 10^{10}\,cm^{-2}$ which corresponds to an excitation of $E \approx 1\,meV$ (a band gap of 1 meV can be obtained by applying back and top gate values of for example $V_{BG} = 0.14\,V$ and $V_{SG} = -0.13\,V$ respectively). At back and split gate values of $V_{BG} = 8\,V$ and $V_{SG} = -7.6\,V$ respectively, which corresponds to a displacement field of $D \approx 0.56\,V/nm$ following Zhang *et al.* [15], we obtain an energy band gap of $E_g \approx 85.4\,meV$, *i.e.* a value much larger than the potential fluctuation.

SUPPLEMENTARY NOTE 3: FABRY-PÉROT INTERFERENCE ANALYSIS

The gate dependence of the conductance reveals multiple oscillation patterns that can be attributed to Fabry-Pérot interferences of different cavities [16–19]. Supplementary Figure 3 shows dG/dV_{BG} as a function of back- and split-gate voltage (V_{BG}, V_{SG}), where the data of the conductance G is numerically differentiated with respect to V_{BG}. Taking into account the size of the cavity and the gate dependence of the interferences, *i.e.* slope and appearance in the gate map, the observed patterns can be unambiguously assigned to the corresponding cavities (see Supplementary Figure 3a, c, e, f). The effective cavity length L can be determined by the relation $\Delta n = 2\sqrt{\pi n}/L$ which follows from the resonance condition $\Delta k \cdot L = \pi$.

In the unipolar regime PPP, pn-junctions arise at the interface of the graphene sheet with the two metallic electrodes. Thus, a cavity is formed between the two outer contacts. At a density $n \approx 2 \cdot 10^{12}\,cm^{-2}$ ($V_{SG} = 0$) the spacing between interference peaks is $\Delta n \approx 5 \cdot 10^{10}\,cm^{-2}$. By using $\Delta n = 2\sqrt{\pi n}/L$, we find an effective cavity length of $L \approx 1\,\mu m$ which is consistent with the geometrical size of the device. Therefore, we can conclude that such Fabry-Pérot interferences indicate ballistic transport on a length scale of at least twice the device length, *i.e.* $\approx 2\,\mu m$.

SUPPLEMENTARY NOTE 4: SUPERCURRENT AND MULTIPLE ANDREEV REFLECTION

Here, we provide additional information on the superconducting properties of our sample. As we have seen, the magnitude of the supercurrent is back-gate tunable, *i.e.* depends on the charge carrier density. While at the charge neutrality point superconductivity is fully suppressed, large supercurrent densities of $\sim 580\,nA\,\mu m^{-1}$ at $n \approx 4 \cdot 10^{12}\,cm^{-2}$ are measured. Furthermore, the supercurrent amplitude is dramatically reduced by the two pn-junctions formed at bilayer graphene-metal contact interfaces when the back-gate tunes the Fermi level in the valence band ($V_{BG} < 0$) (*i.e.* the PPP and PNP regions). The current-voltage characteristics extracted from the PPP region

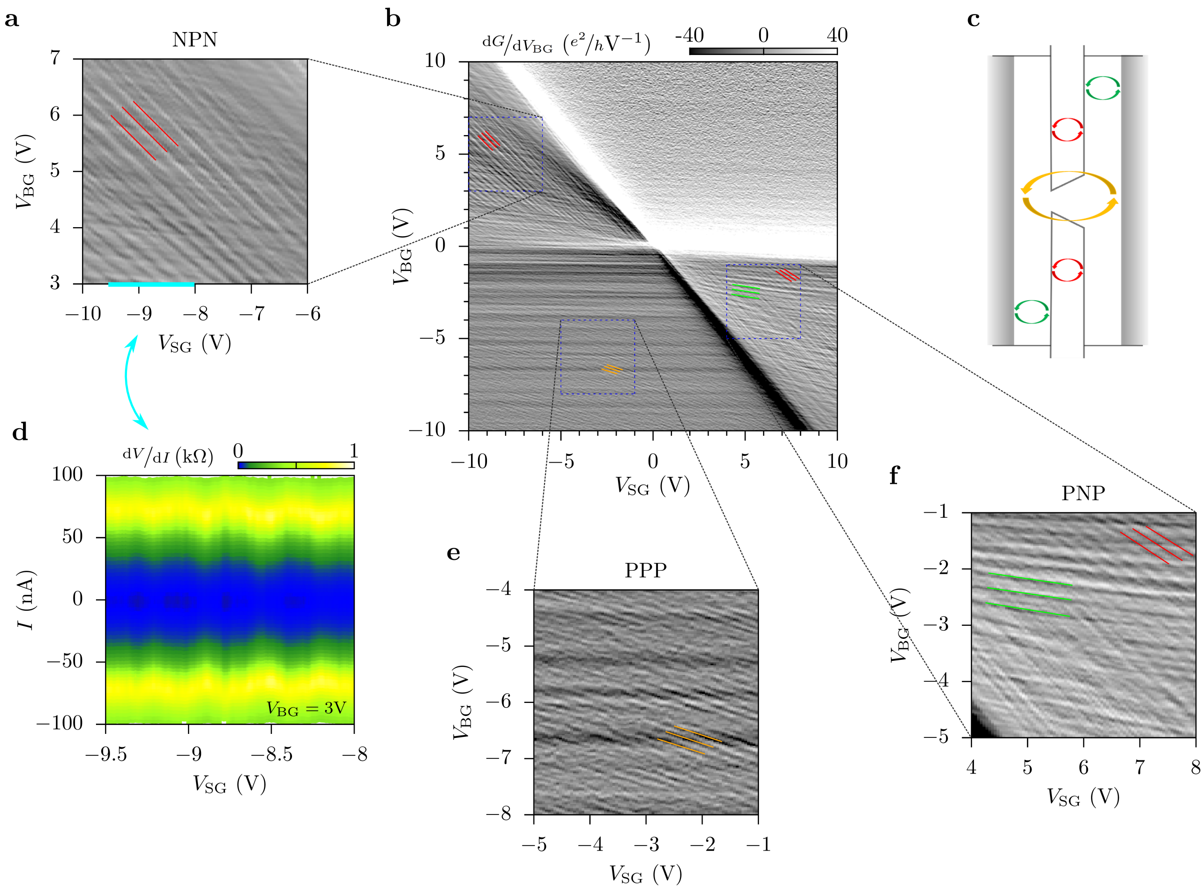

SUPPLEMENTARY FIGURE 3. **Fabry-Pérot resonances. b,** Differentiated conductance $\mathrm{d}G/\mathrm{d}V_{\mathrm{BG}}$ as a function of back- and split-gate voltage ($V_{\mathrm{BG}}, V_{\mathrm{SG}}$) measured at 20 mT (normal state). Fabry-Pérot resonances can be observed in the parts of the map where cavities are formed by pn-junctions. **a, e, f,** Zoom-in on the NPN, PNP and PPP region of the gate map. The visible resonances are highlighted by exemplary lines and schematically represented on panel **c,** where the device can be divided into the corresponding cavities. **d**, differential resistance $\mathrm{d}V/\mathrm{d}I$ *vs* current bias I and split-gate voltage V_{SG} measured along the cyan line of panel **a**. Oscillations of the supercurrent due to the Fabry-Pérot interferences are visible.

show a strong attenuation (by approximately one order of magnitude) compared to the NNN part where no pn-junction is formed (see Supplementary Figure 4a). The product of the critical current and the normal state resistance $I_{\mathrm{c}}R_{\mathrm{n}}$ as a function of the back-gate voltage V_{BG} at $V_{\mathrm{SG}} = 0$ (in blue) and $I_{\mathrm{c}}R_{\mathrm{n}}$ as a function of the split-gate voltage V_{SG} at $V_{\mathrm{BG}} = 8$ V (in red) are displayed in Supplementary Figure 4b. In the absence of voltage applied on the split-gate, a clear asymmetry between hole and electron conduction is visible in the bipolar supercurrent amplitude reflecting the presence of the two pn-junctions formed at the contacts. When the split-gate voltage increases,

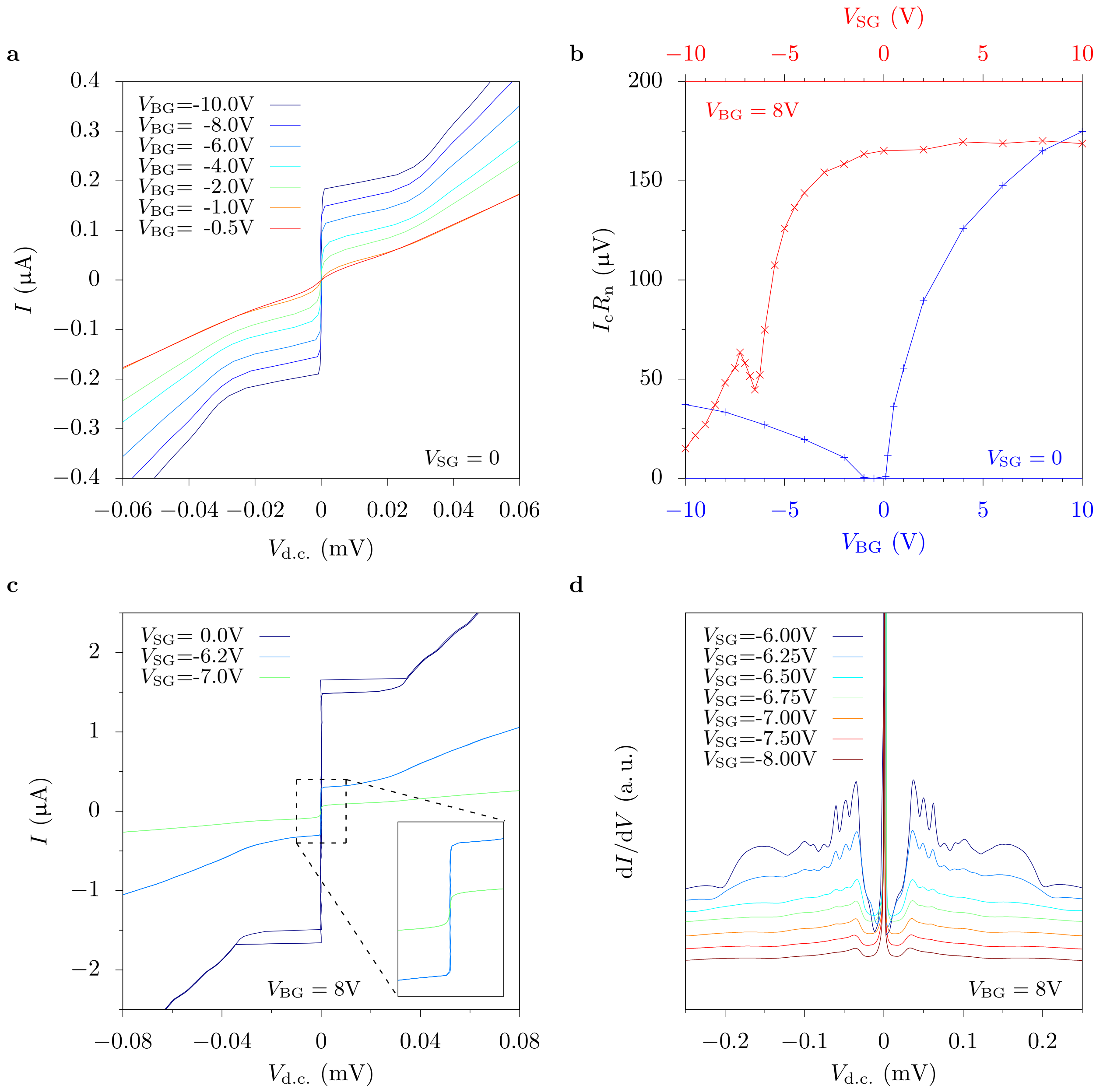

SUPPLEMENTARY FIGURE 4. ***I*-*V* curves, I_cR_n product and multiple Andreev reflection. a,** Series of I-V curves in the p-doped region at $V_{SG} = 0$. **b,** I_cR_n versus V_{BG} at $V_{SG} = 0$ (in blue) and I_cR_n versus V_{SG} at $V_{BG} = 8$ V (in red). **c,** The I-V curves show the behaviour for the three different cases: no split-gate ($V_{SG} = 0$), right before ($V_{SG} = -6.2$ V) and once the constriction is fully developed ($V_{SG} = -7$ V), at $V_{BG} =$ 8 V. A zoom-in of the two curves taken at $V_{SG} = -6.2$ V and -7 V is displayed in the inset, highlighting the absence of hysteresis before and after the formation of the constriction. **d,** Series of differential conductance dI/dV *vs* voltage V_{DC} for various split-gate voltages V_{SG} at $V_{BG} = 8$ V. The curves are shifted for clarity. MAR clearly vanishes with the creation of the 1D constriction.

the I_cR_n product remains stable until the constriction starts to form. Then, the I_cR_n product decreases rapidly. We note that contrary to $I_c(V_{SG})$ which decays monotonically, the I_cR_n product suddenly increases slightly between 6 V and 7 V corresponding to the voltage range of the 2D to 1D transition, before decreasing again as the constriction size shrinks.

	V_{SG}(V)	V_{BG}(V)	n(cm^{-2})	I_c(μA)	I_r(μA)	I_r/I_c	I_cR_n(μV)
NNN	0	10	$\sim 4\times10^{12}$	1.86	1.66	0.9	174.8
	0	8	$\sim 3.2\times10^{12}$	1.66	1.48	0.9	165.1
	0	6	$\sim 2.4\times10^{12}$	1.38	1.25	0.9	147.7
	0	4	$\sim 1.6\times10^{12}$	1.05	0.95	0.9	126
	0	2	$\sim 0.8\times10^{12}$	0.585	0.585	1	89.5
PPP	0	-10	$\sim -4\times10^{12}$	0.185	0.185	1	37.2
	0	-8	$\sim -3.2\times10^{12}$	0.152	0.152	1	33.4
NNN	-6.2	8	$\sim 3.2\times10^{12}$	0.295	0.295	1	56.3
NP_nN	-7	8	$\sim 3.2\times10^{12}$	0.071	0.071	1	58.1

SUPPLEMENTARY TABLE I. **Compilation of some of the superconducting characteristics**. Critical current I_c, the retrapping current I_r, I_r/I_c and I_cR_n product under several gate conditions corresponding to different regions of the gate map (see Fig. 2 of the main text).

The impact of the constriction on the hysteretic behaviour of the Josephson effect is shown in Supplementary Figure 4c. As we can see on the I-V curves (up and back bias sweeps), the hysteresis occurs at sufficiently high charge carrier density and disappears once the constriction

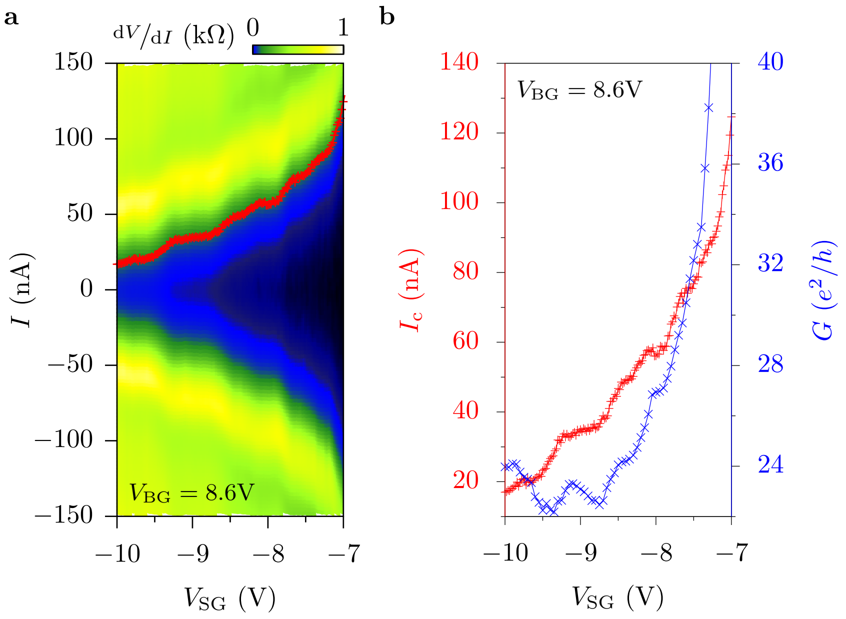

SUPPLEMENTARY FIGURE 5. **Step-like features in the supercurrent and in the normal state conductance**. **a,** Differential resistance $\mathrm{d}V/\mathrm{d}I(V_{\mathrm{SG}}, I)$ map as a function of split-gate voltage V_{SG} and current I zoomed-in on the $\mathrm{NP_nN}$ region, revealing a step-wise reduction of the critical current I_{c} (red curve). **b,** Critical current I_{c} (red) and normal state conductance G (blue) *vs* V_{SG} at $V_{\mathrm{BG}} = 8.6\,\mathrm{V}$.

develops. Supplementary Table I recapitulates the extracted I_{c}, the retrapping current I_{r} and the ratio $I_{\mathrm{r}}/I_{\mathrm{c}}$ in the various regions of the gate map (see Fig. 2 of the main text). Within the RCSJ model [20], the Josephson junction is tuned from underdamped to overdamped. We note that for small n-doping, *i.e.* $n < 1.5 \cdot 10^{12}\,\mathrm{cm}^{-2}$, as well as for p-doping no hysteresis is detected.

Supplementary Figure 4d exhibits the effect of the constriction on multiple Andreev reflection (MAR). MAR appears as peaks in the differential conductance $\mathrm{d}I/\mathrm{d}V$ versus bias voltage V_{DC} and are positioned at $2\Delta/en$ (with $n = 1, 2, 3, 4, ...$ and Δ being the superconducting gap, here estimated at $\sim 100\,\mu\mathrm{eV}$). When the constriction is formed, these subgap features disappear while a finite supercurrent remains detectable. The constriction limits dramatically the possibilities for the reflected quasi-particles to return to the opposite lead, and therefore vanishes the MAR. It is important to note that the disappearance of MAR coincides with the reduction of the supercurrent amplitude and the change in the magneto-interferometric pattern (see Fig. 3d and 4 in the main text).

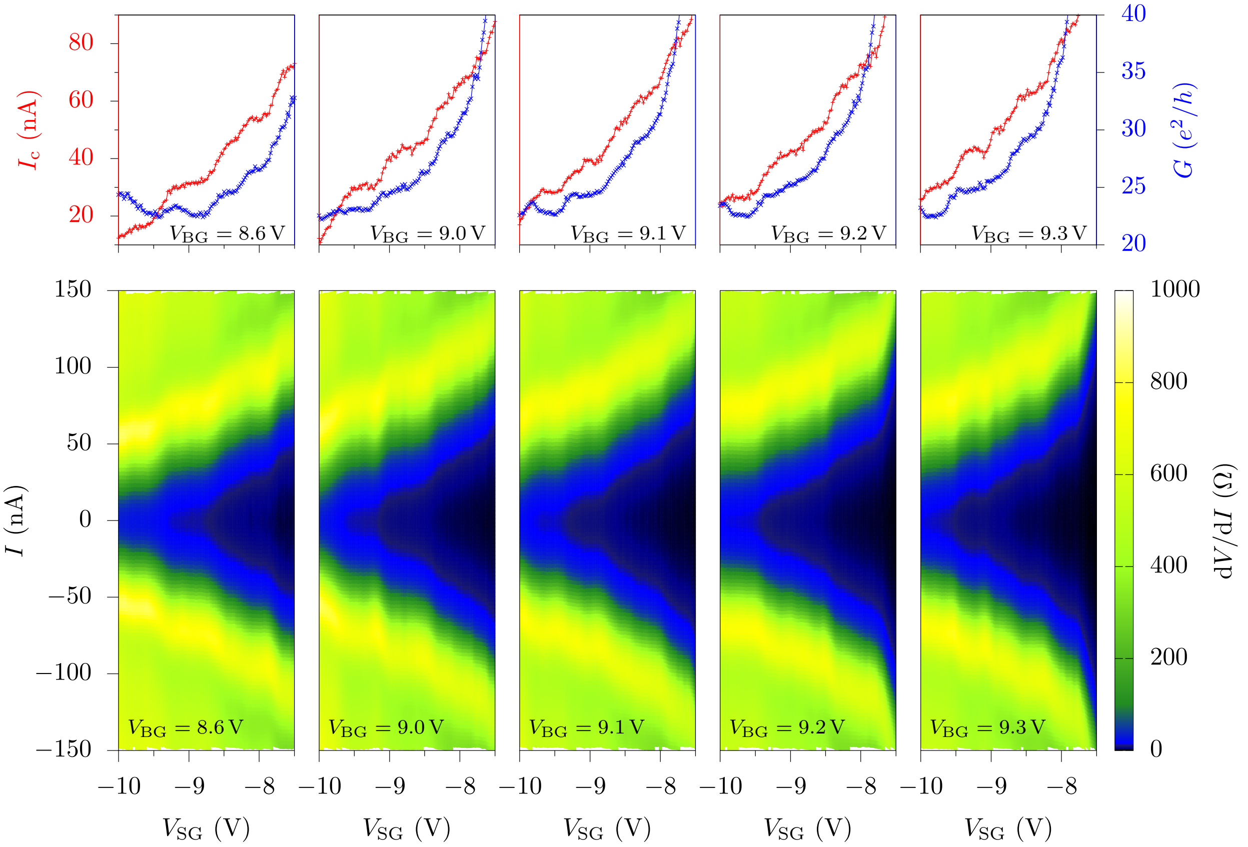

SUPPLEMENTARY FIGURE 6. **Step-like features in the supercurrent under various back-gate conditions**. Critical current I_c and conductance G (top) and differential resistance $\mathrm{d}V/\mathrm{d}I(V_{SG}, I)$ maps (bottom) showing step-like features in the supercurrent for various back-gate voltages, from left to right $V_{BG} = 8.6, 9.0, 9.1, 9.2$ and 9.3 V respectively.

SUPPLEMENTARY NOTE 5: ANY SIGNS OF QUANTIZED SUPERCURRENT?

In our experiments, we clearly observe Fabry-Pérot interferences highlighting ballistic transport of the charge carriers all across the length of the device. Therefore, one might expect to observe quantized conductance while inducing a constriction in a two-dimensional system [21, 22]. This phenomenon has been extensively studied in particular in AlGaAs/GaAs heterostructures [23]. Until now, step-like-features in the conductance have been observed in graphene (both single and bilayers) but none of them quantized in the expected value of $4e^2/h$ [24–33] (the prefactor 4 refers to the spin and valley degeneracy). For our configuration using only one overall back-gate and a local split-gate, both the tuning of the Fermi energy and the opening of the gap cannot be fully independently controlled within the 1D constriction. At V_{BG} and V_{SG} sufficiently large to form the

constriction and confine the supercurrent, the charge carrier density within the constriction (mostly influenced by the back-gate compared to the stray field generated by the split-gate) might appear too large and the confinement not strong enough to clearly form 1D subbands and therefore both quantized conductance and supercurrent appear to be hard to observe. Therefore the picture drawn for more conventional semiconductors might not be applicable [34–36]. In our case we observe features in the normal and superconducting state conductance although non-quantized, here at $V_{\mathrm{BG}} = 8.6\,\mathrm{V}$ (see Supplementary Figure 5a and b). At this back gate value the $\mathrm{NP_nN}$ and $\mathrm{NP_pN}$ border corresponds to $V_{\mathrm{SG}} \sim -8.5\,\mathrm{V}$. As we can see, the minimum conductance reaches $24e^2/h$ corresponding to 6, four times degenerated, opened channels and a resistance of $\sim 1\,\mathrm{k\Omega}$. We note that these features are observable in a large back-gate range (see Supplementary Figure 6). What we define as signs of quantized supercurrent (together with signs of quantized conductance) was measured in our shorter device with w ~65 nm split-gate distance but not in our longer device with a wider constriction (w ~150 nm split-gate distance, not shown here) which showed all properties of confinement (in both amplitude and magneto-interferometric pattern) that we present in this work.

SUPPLEMENTARY NOTE 6: MAGNETIC INTERFERENCE PATTERNS AT $\mathbf{V_{BG} = 4\,V}$

Here we show an additional series of data where the change of the magnetic interferences clearly shows a transition from a beating to a non-beating pattern corresponding to the creation of the 1D constriction. Supplementary Figure 7a displays a series of resistance maps versus current and magnetic field at constant density ($V_{\mathrm{BG}} = 4\,\mathrm{V}$) in a similar fashion as the data taken at higher density presented in the main text. Here, the transition from a beating to a non-beating pattern occurs in a voltage range -3 V < V_{SG} < -3.5 V. In Supplementary Figure 7b, we see the two coloured maps of the critical current I_{c} (left panel) and the critical current normalised with the maximum critical current (at $B = 0$) $I_{\mathrm{c}}^{\mathrm{norm.}}$ (right panel) as a function of magnetic field B and split-gate voltage V_{SG}.

SUPPLEMENTARY NOTE 7: ANALYTICAL MODEL: LONG JUNCTION

We calculate the Josephson current $J(\chi)$ through the sample as a function of the superconducting phase difference $\chi = \chi_2 - \chi_1$ in the presence of magnetic field B, using the quasiclassical

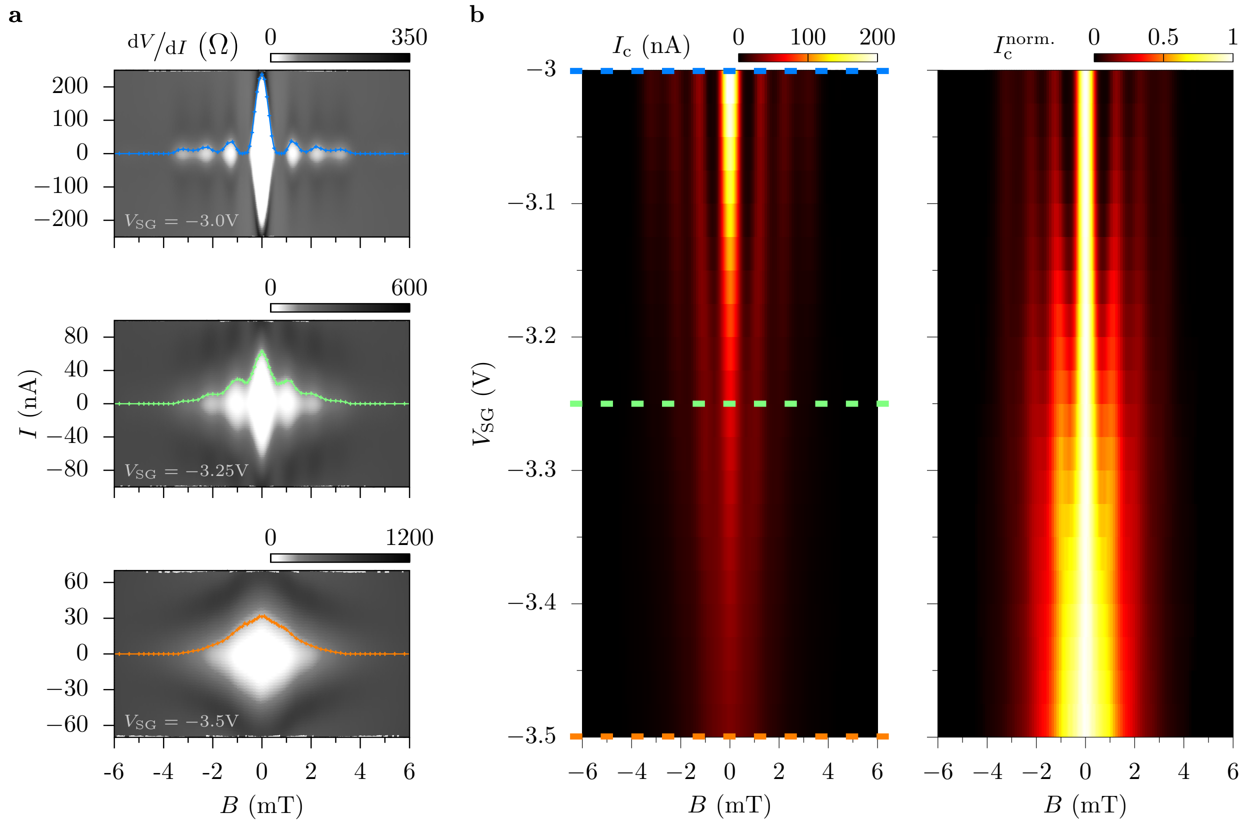

SUPPLEMENTARY FIGURE 7. **Magnetic interferometry study of the transition from 2D to 1D confinement of the supercurrent at V_{BG} = 4 V. a,** Grey-scale maps of the differential resistance dV/dI versus bias current I and magnetic field B measured at $V_{SG} = -3$ V, $V_{SG} = -3.25$ V and $V_{SG} = -3.5$ V. The coloured doted lines correspond to the extracted I_c. **b,** Critical current I_c (left panel) and normalised critical current $I_c^{norm.}$ (right panel) mapped as a function of magnetic field B and split-gate voltage V_{SG}. The coloured dashed lines correspond to side-gate values where the $dV/dI(B, I)$ maps were measured in panels **a**.

approach developed in Refs. [37, 38] and [39]. The essence of this approach lies in expressing the superconducting current density in terms of quasiclassical trajectories connecting the superconducting leads. These paths can be viewed as electron-hole "tubes" of width $\sim \lambda_F$, resulting from the Andreev reflection at the NS interfaces and corresponding to Andreev bound states. Each path is associated with the partial contribution to the Josephson current that depends on the positions of end points (y_1, y_2 in Supplementary Figure 8) and on magnetic field that enters through the Aharonov-Bohm phase.

The magnetic interference pattern is obtained after the summation over all paths and maximiz-

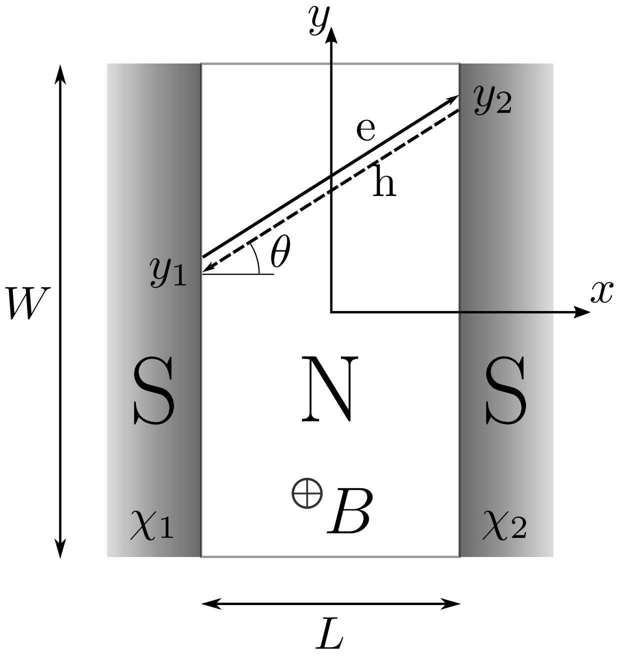

SUPPLEMENTARY FIGURE 8. **Schematics of the SNS junction.** Schematics of the SNS setup at zero split-gate voltage.

ing the Josephson current with respect to χ:

$$I_c(\phi) = \max_\chi\{J(\chi, \phi)\}. \tag{1}$$

Here $\phi = \Phi/\Phi_0 = BWL/\Phi_0$ is the dimensionless magnetic flux through the sample in units of $\Phi_0 = \pi\hbar c/e$.

Since the thermal length $L_\mathcal{T} \sim \hbar v/(k_B\mathcal{T})$ for the experimental temperature is much larger than L, we set $\mathcal{T} = 0$. Following Ref. [37], we write the Josephson current for a long junction ($L \gg \xi$, where ξ is the superconducting coherence length) as an integral over the end points of the Andreev tubes (Supplementary Figure 8)

$$J(\chi, \phi) = \frac{2ev_F}{\pi\lambda_F L^2}\iint_{-W/2}^{W/2} dy_1 dy_2 \frac{\mathcal{J}[\tilde{\chi}(y_1, y_2)]}{\left[1 + \left(\frac{y_1 - y_2}{L}\right)^2\right]^2}, \tag{2}$$

where $\mathcal{J}$ is the dimensionless partial Josephson current associated with points y_1 and y_2 and $\tilde{\chi}(y_1, y_2)$ is the effective phase difference in magnetic field. Each straight trajectory connecting points y_1 and y_2 is characterised by angle θ between the trajectory and x-axis, $\tan\theta = (y_2 - y_1)/L$.

In the long-junction limit, the partial current is given by

$$\mathcal{J}(\chi) = \sum_{k=1}^{\infty} \frac{(-1)^{k+1} T^k}{k} \sin(k\chi) = \mathrm{Im}\left[\ln\left(1 + T e^{i\chi}\right)\right], \quad \xi \ll L, \tag{3}$$

where we introduced the transmission probability $T \leq 1$. For $T \ll 1$ only the $k = 1$ term is important, leading to

$$\mathcal{J}(\chi) \simeq T \sin\chi, \quad T \ll 1, \tag{4}$$

which is the conventional Josephson relation (also valid for $T \ll 1$ in the short-junction limit).

To include the magnetic field into the consideration, it is convenient to choose the x dependent gauge for the vector potential as in Ref. [39] (assuming small London penetration length for superconducting leads):

$$\mathbf{A} = A_y \mathbf{e}_y, \quad A_y = \begin{cases} -Bx, & -L/2 \leq x \leq L/2, \\ -\frac{1}{2} BL \operatorname{sign} x, & |x| > L/2. \end{cases} \tag{5}$$

For such a vector potential, the phase difference due to the magnetic phase acquired on straight trajectories connecting the two interfaces vanishes. At the same time, the superconducting phases at the interfaces become functions of y [39]:

$$\tilde{\chi}(y_1, y_2) = \chi - \frac{\pi\phi(y_1 + y_2)}{W}. \tag{6}$$

Let us now employ the formalism described above to the QPC setup. For simplicity, we neglect the geometrical width of the infinitely strong barriers. We assume that the setup is symmetric, *i.e.*, the QPC is located at $x = 0$ and $y = 0$. The QPC has the width which is of the order of (or smaller than) λ_F and hence is approximately characterised by an isotropic transmission probability. For low transmission $T_0 \ll 1$, one can retain only the conventional first harmonics in the partial Josephson currents, Supplementary Equation (4). This implies that under these assumptions, the shape of the magnetic interference pattern is, in fact, not sensitive to the relation between ξ and L (long *vs.* short junction).

In terms of the quasiclassical trajectories, the only possible trajectory connecting the points y_i and y_f at the opposite interfaces should pass through the QPC (here we discard the boundary scattering). The trajectory is now parameterised by the two angles: θ_i, corresponding to the velocity in the region $-L/2 < x < 0$, and θ_f in the region $0 < x < L/2$ after transmission through the QPC. These angles satisfy the relations:

$$\tan\theta_i = -\frac{2y_i}{L}, \quad \tan\theta_f = \frac{2y_f}{L}. \tag{7}$$

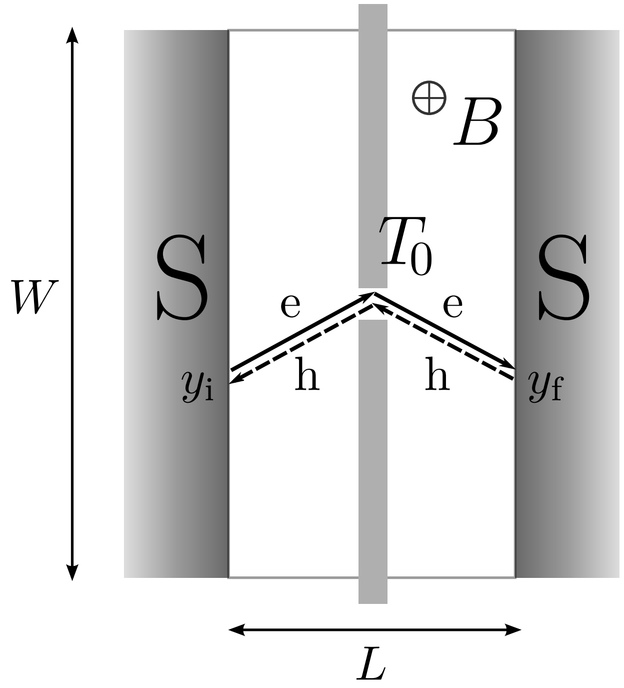

SUPPLEMENTARY FIGURE 9. **Schematics of the SNS junction with QPC.**QPC setup with split-gate.

With the gauge (5), the magnetic phase acquired within the sample reads:

$$\frac{2\pi}{\Phi_0}\int \mathrm{d}\mathbf{l}\cdot\mathbf{A} = -\frac{\pi B}{\Phi_0}\left(\frac{L}{2}\right)^2(-\tan\theta_\mathrm{i}+\tan\theta_\mathrm{f}) = -\frac{\pi\phi(y_\mathrm{i}+y_\mathrm{f})}{2W}. \tag{8}$$

The total phase difference is given by the difference of the magnetic phase (8) and the superconducting phase difference in the presence of magnetic field [Supplementary Equation (6)]:

$$\tilde{\chi}(y_\mathrm{i}, y_\mathrm{f}) = \chi - \frac{\pi\phi}{2W}(y_\mathrm{i}+y_\mathrm{f}). \tag{9}$$

As compared to the case without the QPC, the phase difference induced by magnetic field is reduced by a factor of two. Indeed, the area between the Andreev tube connecting the points y_i and y_f and the line $y = 0$ is twice smaller for the trajectory going through the point $x = 0$, $y = 0$ than for the straight line.

The normalised critical current now reads

$$\frac{I_\mathrm{c}(\phi)}{I_\mathrm{c}(0)} = \frac{\max_\chi \int \mathrm{d}\theta_\mathrm{i}\cos^2\theta_\mathrm{i}\int \mathrm{d}\theta_\mathrm{f}\cos\theta_\mathrm{f}\mathcal{J}(\tilde{\chi}(\theta_\mathrm{i},\theta_\mathrm{f}))}{\max_\chi \int \mathrm{d}\theta_\mathrm{i}\cos^2\theta_\mathrm{i}\int \mathrm{d}\theta_\mathrm{f}\cos\theta_\mathrm{f}\mathcal{J}(\chi)}. \tag{10}$$

In the limit of small transmission probability $T_0 \ll 1$ we use Supplementary Equation (4) for the partial Josephson current. The integrations over θ_i and θ_f then separate and the normalised critical current can be written as

$$\frac{I_\mathrm{c}(\phi)}{I_\mathrm{c}(0)} = \frac{\mathcal{I}_2(\phi)\mathcal{I}_{3/2}(\phi)}{\mathcal{I}_2(0)\mathcal{I}_{3/2}(0)}. \tag{11}$$

Here, the integrals $\mathcal{I}$ are defined as

$$\mathcal{I}_k(\phi) = \frac{2}{L}\int_{-W/2}^{+W/2} \mathrm{d}y \frac{\cos\left(\frac{\pi\phi y}{2W}\right)}{\left[1+\left(\frac{2y}{L}\right)^2\right]^k}. \tag{12}$$

At $\phi = 0$ we get

$$\mathcal{I}_2(0)\mathcal{I}_{3/2}(0) = \frac{L}{\sqrt{L^2+W^2}}\arctan\frac{W}{L} + \frac{L^2 W}{(L^2+W^2)^{3/2}}. \tag{13}$$

The parabolic asymptotics of the critical current at small ϕ is found by expanding the cosine factors in the numerator:

$$\frac{I_\mathrm{c}(\phi)}{I_\mathrm{c0}} \simeq 1 - \frac{\pi^2\phi^2}{32} f_0(W/L) \tag{14}$$

$$f_0(x) = \frac{\sqrt{x^2+1}\log\left(\sqrt{x^2+1}+x\right)}{x} - \frac{x}{x+(x^2+1)\arctan(x)} \tag{15}$$

In the limit of high fields, $\phi \to \infty$, we extend the integration in Supplementary Equation (12) over y_i and y_f to $\pm\infty$ and obtain

$$\frac{I_\mathrm{c}(\phi)}{I_\mathrm{c0}} \simeq \frac{\pi^{3/2}\left(1+x^2\right)^{3/2}}{8x^2\left[x+(1+x^2)\arctan x\right]}\left(\frac{\pi\phi}{2x}\right)^{3/2}\exp\left(-\frac{\pi\phi}{2x}\right). \tag{16}$$

The evaluation of the integrals in Supplementary Equation (10) with a transmission probability of $T_0 = 1/2$ in the whole range of magnetic fields yields the curve for the normalised critical current shown in Fig. 5b of the main text. Interestingly, the result for $T_0 = 1/2$ is almost indistinguishable from the analytical result for $T_0 \ll 1$. The width of the analytical curve was fitted to the experimental one thus accounting for the actual geometry and those factors (*e.g.*, the finite width of the barriers and the finite magnetic penetration depth) that were neglected in the simplified model.

Supplementary Note 8: Numerical model: Short junction

We study a supercurrent through a superconductor–bilayer-graphene–superconductor (SBLGS) Josephson junction (JJ) in the presence of top split gates in the experimental geometry, as shown in Supplementary Figure 10. In particular, we investigate the orbital effects of the magnetic field which is applied perpendicular (say in z-direction) to the junction in the normal region. For numerical simulations, we use a Landau gauge such that the vector potential is given by $\mathbf{A} = (-By\hat{x}, 0, 0)$.

Here, we explain the formalism used for calculating the supercurrent numerically. We restrict ourselves to the short-junction limit and use the scattering matrix approach. For transport below

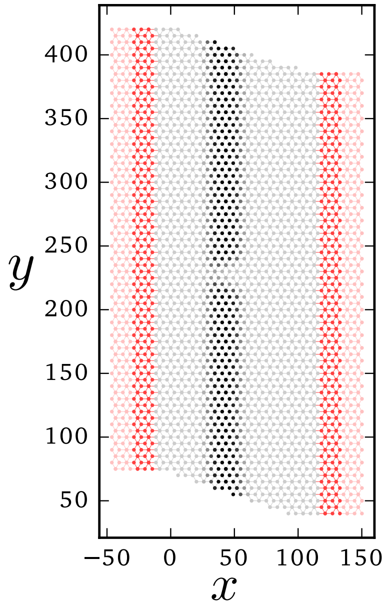

SUPPLEMENTARY FIGURE 10. **Illustration of the setup used for the simulations.** The darker region represents the QPC area while the red strips represent the leads attached to the scattering region. The scales of the x- and y-axis are defined in terms of the tight-binding lattice constant.

the superconducting energy gap, an electron incident on the superconducting interface is Andreev reflected as a hole, which results in emergence of Andreev bound states. To calculate the spectrum of these bound states, we use the two types of scattering matrices. The matrix s_N, which is due to the normal region and is block diagonal in electron and hole space, is defined as

$$s_{\mathrm{N}}(\epsilon) = \begin{bmatrix} s(\epsilon) & 0 \\ 0 & s^*(-\epsilon) \end{bmatrix}, \tag{17}$$

while the other matrix s_{A} is due to Andreev reflections and is block off-diagonal as it couples electrons with holes:

$$s_{\mathrm{A}}(\epsilon) = \alpha(\epsilon) \begin{bmatrix} 0 & r_{\mathrm{A}}^* \\ r_{\mathrm{A}} & 0 \end{bmatrix}, \tag{18}$$

with

$$\alpha(\epsilon) = \exp[-i \arccos(\epsilon/\Delta)]$$

and r_{A} a diagonal matrix

$$r_{\mathrm{A}} = \begin{bmatrix} ie^{i\chi/2}\mathbf{1}_{n_1} & 0 \\ 0 & ie^{-i\chi/2}\mathbf{1}_{n_2} \end{bmatrix}. \tag{19}$$

In general, these two matrices depend on the energy ϵ of the state. However, in the short-junction limit, we have $s(\epsilon) \approx s(-\epsilon) \approx s(0) \equiv s$. The condition on these scattering matrices to have a bound state is given in Ref. [40]:

$$s_{\mathrm{A}}(\epsilon)s_{\mathrm{N}}(\epsilon)\Psi_{\mathrm{in}} = \Psi_{\mathrm{in}}, \tag{20}$$

where $\Psi_{\mathrm{in}} = (\Psi^e_{\mathrm{in}}, \Psi^h_{\mathrm{in}})$ is a vector of complex coefficients describing a wave incident on the junction in the basis of modes incoming from the superconducting leads into the normal region. By using the individual matrices, we arrive at the following eigenvalue problem:

$$\begin{bmatrix} s^{\dagger} & 0 \\ 0 & s^T \end{bmatrix}\begin{bmatrix} 0 & r^*_{\mathrm{A}} \\ r_{\mathrm{A}} & 0 \end{bmatrix}\Psi_{\mathrm{in}} = \alpha\Psi_{\mathrm{in}}. \tag{21}$$

By applying Joukowsky transform, we can map the above eigenvalue problem from α to ϵ/Δ [41]:

$$\begin{bmatrix} 0 & -iA^{\dagger} \\ iA & 0 \end{bmatrix}\Psi_{\mathrm{in}} = \frac{\epsilon}{\Delta}\Psi_{\mathrm{in}}, \tag{22}$$

where

$$A \equiv \frac{1}{2}(r_{\mathrm{A}}s - s^T r_{\mathrm{A}}).$$

For only electron states, we can write from the above equation

$$A^{\dagger}A\Psi^e_{\mathrm{in}} = \frac{\epsilon^2}{\Delta^2}\Psi^e_{\mathrm{in}}. \tag{23}$$

In the limit of first order perturbation theory, we can write an expression for the differential of bound state energy with respect to the superconducting phase as

$$\frac{\mathrm{d}\epsilon}{\mathrm{d}\chi} = \frac{\Delta^2}{2\epsilon}\frac{\langle\Psi_{\mathrm{in}}|\,\frac{\mathrm{d}(A^{\dagger}A)}{\mathrm{d}\chi}\,|\Psi_{\mathrm{in}}\rangle}{\langle\Psi_{\mathrm{in}}|\,\Psi_{\mathrm{in}}\rangle}. \tag{24}$$

The advantage of having the above expression is that one do not need to take a numerical differentiation of bound state energy over χ. Instead we just solve an eigenvalue problem for this differential. As a result, we obtain the Josephson current as

$$J(\chi) = -\frac{2e}{\hbar}\sum_p \tanh(\epsilon_p/2k_{\mathrm{B}}\mathcal{T})\frac{\mathrm{d}\epsilon_p}{\mathrm{d}\chi}. \tag{25}$$

The critical current I_{c} is then obtained from Supplementary Equation (1).

The tight-binding Hamiltonian for the normal scattering region can be written as

$$H = -t\sum_{i,j,m} e^{\iota\phi_{ij}}a^{\dagger}_{mi}b_{mj} - \gamma_1\sum_j a^{\dagger}_{1j}b_{2j} - \sum_{i,m}(\mu_i - (-1)^m\delta_i)(a^{\dagger}_{mi}a_{mi} + b^{\dagger}_{mi}b_{mi}) + H.c., \tag{26}$$

with the magnetic phases $\phi_{i,j} = \frac{2\pi e}{h}\int_i^j \mathbf{A}\cdot d\mathbf{r}$. The indices i, j corresponds to the lattice sites, whereas $m = 1, 2$ denote the two layers of the BLG. The operators $a_{mi}, a_{mi}^{\dagger}$ ($b_{mi}, b_{mi}^{\dagger}$) are the annihilation and creation operators for electrons at site i in sublattice A_m (B_m), respectively. The parameters t and γ_1 are the intralayer and interlayer (between dimer sites) hopping constants, respectively, whereas μ and δ correspond to the onsite energies given by

$$\mu_i = (\varphi_{\mathrm{BG}} + u_{\mathrm{i}}\varphi_{\mathrm{SG}})/2, \tag{27}$$

and

$$\delta_i = -(u_i\varphi_{\mathrm{SG}} - \varphi_{\mathrm{BG}})/\eta. \tag{28}$$

Here u_i defines the gated region such that

$$u_i = \begin{cases} 1 & \text{if } i \text{ is inside gated region} \\ 0 & \text{if } i \text{ is outside gated region} \end{cases} \tag{29}$$

and η is the numerical factor accounting for the geometry of the setup in the direction perpendicular to the graphene plane. In what follows, we choose $\eta = 2.5$.

In the above equations, φ_{BG} and φ_{SG} represent the strengths of the on-site potentials introduced by the back gate and top split gate, respectively. The corresponding Bogoliubov-De Gennes Hamiltonian for the SBLGS junction reads:

$$H_{\mathrm{BdG}} = \begin{pmatrix} H & \Delta \\ \Delta^* & -H^* \end{pmatrix}, \tag{30}$$

where Δ is a step-like function given by

$$\Delta = \begin{cases} \Delta_0 e^{i\chi_1} & \text{left lead} \\ \Delta_0 e^{i\chi_2} & \text{right lead} \\ 0 & \text{scattering region} \end{cases} . \tag{31}$$

To calculate the scattering matrix s_{N} introduced in the previous section, we use Kwant [42] where we discretise Supplementary Equation (26) on a honeycomb lattice. To verify our model, we fit the experimental data at zero split-gate voltage Supplementary Figure 11. The corresponding magnetic interference pattern appears quite distorted compared to a *sinc* function of a regular, short and wide rectangular junction [20]. The fitting is done by rescaling both axes to match the experimental plot using a junction area of $\sim 4.60\cdot 10^{-12}$ m^2 (corresponding to $\lambda_{\mathrm{L}} = 245$ nm, close to

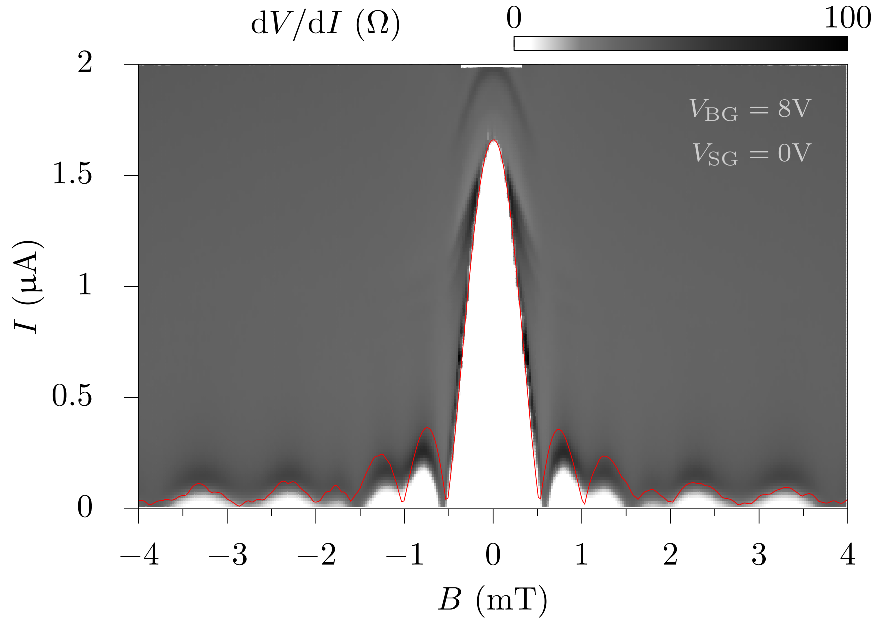

SUPPLEMENTARY FIGURE 11. **Magneto-interference pattern of the 2D system.** Fit (red curve) of experimental magnetic interference pattern $\mathrm{d}V/\mathrm{d}I(B, I)$ at zero split-gate voltage and $V_{\mathrm{BG}} = 8$ V (grey-scale map).

the value extracted from the analytical model $\lambda_{\mathrm{L}} = 275$ nm). It shows that the missing/suppressed lobs of the experimental magnetic interference pattern at zero split-gate voltage match the ones obtained from simulation at some finite strength of the potential in the gated area ($\varphi_{\mathrm{SG}}/t = -0.024$), which may indicate the presence of a small but non-zero potential in the gated region in our experiments. This potential can result from the charge redistribution in the metallic top-gate which is induced by the electrostatic interaction between electrons in the gate and in bilayer graphene.

Here, we describe the parameters used in the simulations displayed in Fig. 5c of the main text. We take $\varphi_{\mathrm{BG}} = 0.2t$, $\gamma_1 = 0.4t$ and vary φ_{SG} starting from the “open” regime (ungapped BLG in gated region) to the “closed” regime (gapped BLG in the gated region). The numerical calculations are performed in the short-junction limit and at finite temperature $k_{\mathrm{B}}\mathcal{T} = \Delta_0/20$.

SUPPLEMENTARY NOTE 9: EFFECT OF THE EDGE CURRENTS ON THE MAGNETO-INTERFEROMETRIC PATTERN

In our experiments, the opening of a band gap by using electrostatic gating is crucial to confine the charge carriers. However, mainly two mechanisms may prevent the confinement [43–50].

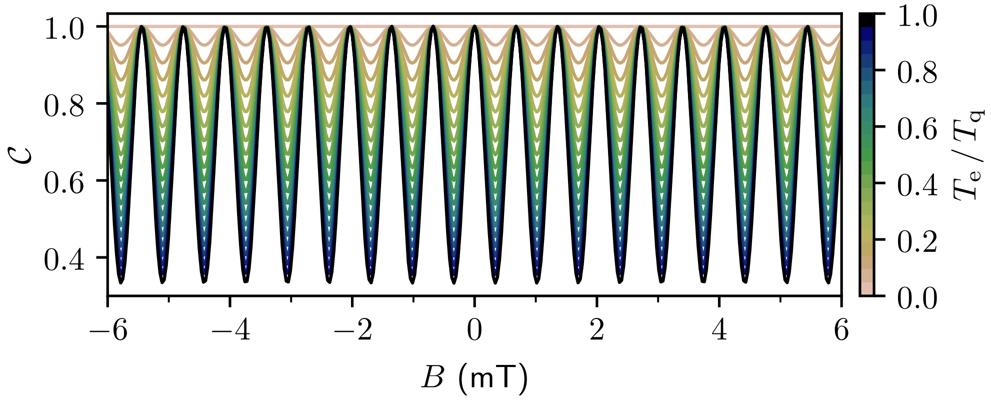

SUPPLEMENTARY FIGURE 12. **Edge current simulations. a.** Correction factor C versus magnetic field B for different values of the transmission coefficient ratio T_e/T_q.

First, stacking defaults which may appear in bilayer graphene and induce topological currents at the boundaries between two domains [46, 47] (*i.e.* between AB and BA stacking domains, along the domain walls). Second, the edge states, which have been predicted to appear in gapped single and bilayer graphene [43, 44], may occur near the charge neutrality point [48–50]. Here we show that if extra current contributes to the total current, *i.e.* other than the current flowing through the constriction, one should be able to detect it via magneto-interferometry.

Following the analytical model presented in Supplementary Note 7, we have extended our calculations by including the possibilities for additional current to flow via the edge of the device as displayed in Fig. 6a of the main text. We use the evaluated integrals within the limit conditions of small transmission $T_0 \ll 1$ and small values of ϕ (Supplementary Equation (14)) and $\phi \to \infty$ (Supplementary Equation (16)) times a correction factor C which reads as follows:

$$C = \frac{|T_q + T_e \cos(\pi\phi)/2|}{(T_q + T_e/2)}, \tag{32}$$

where T_q corresponds to the transmission coefficient of the QPC, T_e is the transmission coefficient of the edge channel, and ϕ is the dimensionless flux (flux in units of Φ_0).

Supplementary Figure 12 shows the variation of the correction factor C as a function of B for various values of the transmission coefficient ratio T_e/T_q. We note that C develops a significant amplitude modulation as $T_e/T_q \to 1$. So the more the edge states are prominent, the larger the variation of C. As T_e raises, the modulation of C increases and C reaches zero at $T_e/2 \sim T_q$. When $T_e \gg T_q$, the correction factor C displays a pattern resembling to the one of a SQUID as expected. We have applied the correction factor to our model and estimated the effect of the presence of edge

contact on the magneto-interference pattern as shown in Fig. 6 of the main text.

SUPPLEMENTARY REFERENCES:

[1] Wang, L., Meric, I., Huang, P.Y., Gao, Q., Gao, Y., Tran, H., Taniguchi, T., Watanabe, K., Campos, L.M., Muller, D.A., Guo, J., Kim, P., Hone, J., Shepard, K.L., & Dean, C.R. One-dimensional electrical contact to a two-dimensional material. *Science* **342**, 614-617 (2013).

[2] Blake, P., Hill, E.W., Castro Neto, A.H., Novoselov, K.S., Jiang, D., Yang, R., Booth, T.J., & Geim, A.K. Making graphene visible. *Appl. Phys. Lett.* **91**, 063124 (2007).

[3] Ferrari, A.C., Meyer, J.C., Scardaci, V., Casiraghi, C., Lazzeri, M., Mauri, F., Piscanec, S., Jiang, D., Novoselov, K.S., Roth, S., & Geim, A.K. Raman spectrum of graphene and graphene layers. *Phys. Rev. Lett.* **97**, 187401 (2006)

[4] Benz, C., Thürmer, M., Wu, F., Ben Aziza, Z., Mohrmann, J., von Löhneysen, H., Watanabe, K., Taniguchi, T., & Danneau, R. Graphene on boron nitride microwave transistors driven by graphene nanoribbon back-gates. *Appl. Phys. Lett.* **102**, 033505 (2013).

[5] Pallecchi, E., Benz, C., Betz, A.C., von Löhneysen, H., Plaçais, B., & Danneau, R. Graphene microwave transistors on sapphire substrates. *Appl. Phys. Lett.* **99**, 113502 (2011).

[6] Steinberg, K., Scheffler, M. & Dressel, M. Quasiparticle response of superconducting aluminum to electromagnetic radiation. *Phys. Rev. B* **77**, 214517 (2008).

[7] Du, X., Skachko, I., Barker, A. & Andrei, E. Y. Approaching ballistic transport in suspended graphene. *Nat. Nanotech.* **3**, 491-495 (2008).

[8] Abanin, D.A. & Levitov, L.S. Conformal invariance and shape-dependent conductance of graphene samples. *Phys. Rev. B* **78**, 035416 (2008).

[9] Williams, J.R., Abanin, D.A., DiCarlo, L., Levitov, L.S., & Marcus, C.M. Quantum Hall conductance of two-terminal graphene devices. *Phys. Rev. B* **80**, 045408 (2009).

[10] Du, X., Skachko, I., Duerr, F., Luican, A. & Andrei, E.Y. Fractional quantum Hall effect and insulating phase of Dirac electrons in graphene. *Nature* **462**, 192-195 (2009).

[11] Bolotin, K.I., Ghahari, F., Shulman, M. D., Stormer, H. L. & Kim, P. Observation of the fractional quantum Hall effect in graphene. *Nature* **462**, 196-199 (2009).

[12] Skachko, I., Du, X., Duerr, F., Luican, A., Abanin, D.A., Levitov, L.S. & Andrei, E.Y. Fractional quantum Hall effect in suspended graphene probed with two-terminal measurements. *Phil. Trans. R. Soc. A* **368**, 5403-5416 (2010).

[13] McCann, E. Asymmetry gap in the electronic band structure of bilayer graphene. *Phys. Rev. B* **74**, 161403 (2006).

[14] McCann, E. & Koshino, M. The electronic properties of bilayer graphene. *Rep. Prog. Phys.* **76**, 056503 (2013).

[15] Zhang, Y. Tang, T.-T., Girit, C., Hao, Z., Martin, M.C., Zettl, A., Crommie, M.F., Shen, Y.R. & Wang, F. Direct observation of a widely tunable bandgap in bilayer graphene. *Nature* **459**, 820-823 (2009).

[16] Shytov, A.V., Rudner, M.S. & Levitov, L.S. Klein backscattering and Fabry-Pérot interference in graphene heterojunctions. *Phys. Rev. Lett.* **101**, 156804 (2008).

[17] Young, A.F., Kim, P. Quantum interference and Klein tunnelling in graphene heterojunctions. *Nat. Phys.* **5**, 222-226 (2009).

[18] Rickhaus, P., Maurand, R., Liu, M.-H., Weiss, M., Richter, K. & Schönenberger, C. Ballistic interferences in suspended graphene. *Nat. Commun.* **4**, 2342 (2013).

[19] Varlet, A., Liu, M.-H., Krueckl, V., Bischoff, D., Simonet, P., Watanabe, K., Taniguchi, T., Richter, K., Ensslin, K. & Ihn, T. Fabry-Pérot interference in gapped bilayer graphene with broken anti-Klein tunneling. *Phys. Rev. Lett.* **113**, 116601 (2014).

[20] Tinkham, M. *Introduction to Superconductivity* (Courier Dover, 2012).

[21] van Wees, B.J., van Houten, H., Beenakker, C.W.J., Williamson, J.G., Kouwenhoven, L.P., van der Marel, D., & Foxon, C.T. Quantized conductance of point contacts in a two-dimensional electron gas. *Phys. Rev. Lett.* **60**, 848-850 (1988).

[22] Wharam, D.A., Thornton, T.J., Newbury, R., Pepper, M., Ahmed, H., Frost, J.E.F., Hasko, D.G., Peacock, D.C., Ritchie, D.A. & Jones, G.A.C. One-dimensional transport and the quantisation of the ballistic resistance. *J. Phys. C* **21**, L209 (1988).

[23] Datta, S. *Electronic Transport in Mesoscopic Systems* (Cambridge University Press, 1995).

[24] Lin, Y.-M. Perebeinos, V., Chen, Z. & Avouris P. Electrical observation of subband formation in graphene nanoribbons. *Phys. Rev. B* **78**, 161409(R) (2008).

[25] Lian, C., Tahy, K., Fang, T., Li, G., G.X., Huili & Jenaa, D. Quantum transport in graphene nanoribbons patterned by metal masks. *Appl. Phys. Lett.* **96**, 103109 (2010).

[26] Tombros, N., Veligura, A., Junesch, J., Guimarães, M.H.D. Vera-Marun, I.J., Jonkman, H.T. & van

Wees, B.J. Quantized conductance of a suspended graphene nanoconstriction. *Nat. Phys.* **7**, 697-700 (2011).

[27] Allen, M.T., Martin, J., & Yacoby, A. Gate-defined quantum confinement in suspended bilayer graphene. *Nat. Commun.* **3**, 934 (2012).

[28] Goossens, A.M., Driessen, S.C.M., Baart, T.A., Watanabe, K., Taniguchi, T. & Vandersypen, L.M.K. Gate-defined confinement in bilayer graphene-hexagonal boron nitride hybrid devices. *Nano Lett.* **12**, 4656-4660 (2012).

[29] Dröscher, S., Barraud, C., Watanabe, K., Taniguchi, T., Ihn, T. & Ensslin, K. Electron flow in split-gated bilayer graphene. *New J. Phys.* **14**, 103007 (2012).

[30] Terrés, B., Chizhova, L.A., Libisch, F., Peiro, J., Jörger, Engels, D.S., Girschik, A., Watanabe, K., Taniguchi, T., Rotkin, S.V., Burgdörfer, J. & Stampfer, C. Size quantization of Dirac fermions in graphene constrictions. *Nat. Commun.* **7**, 11528 (2016).

[31] Li, J., Wang, K., McFaul, K.J., Zern, Z., Ren, Y., Watanabe, K., Taniguchi, T., Qiao, Z., & Zhu J. Gate-controlled topological conducting channels in bilayer graphene. *Nat. Nanotech.* **11**, 1060-1065 (2016).

[32] Somanchi, S., Terrés, B., Peiro, J., Staggenborg, M., Watanabe, K., Taniguchi, T., Beschoten, B. & Stampfer, C. From diffusive to ballistic transport in etched graphene constrictions and nanoribbons. *Ann. Phys.* **529**, 1700082 (2017).

[33] Overweg, H., Eggimann, H., Chen, X., Slizovskiy, S., Eich, M., Pisoni, R., Lee, Y., Rickhaus, P., Watanabe, K., Taniguchi, T., Fal'ko, V., Ihn, T. & Ensslin, K. Electrostatically induced quantum point contact in bilayer graphene. *Nano Lett.* **18**, 553-559 (2018).

[34] Furusaki, A., Takayanagi, H. & Tsukada, M. Theory of quantum conduction of supercurrent through a constriction. *Phys. Rev. Lett.* **67**, 132-135 (1991).

[35] Furusaki, A. Josephson effect of the superconducting quantum point contact. *Phys. Rev. B* **45**, 10563-10575 (1992).

[36] Takayanagi, H., Akazaki, T. & Nitta, J. Observation of maximum supercurrent quantization in a superconducting quantum point contact. *Phys. Rev. Lett.* **75**, 3533-3536 (1995).

[37] Barzykin, V. & Zagoskin, A.N. Coherent transport and nonlocality in mesoscopic SNS junctions: anomalous magnetic interference patterns. *Superlatt. Microstruc.* **25**, 797 (1999).

[38] Sheehy, D.E. & Zagoskin, A.M. Theory of anomalous magnetic interference pattern in mesoscopic superconducting/normal/superconducting Josephson junctions. *Phys. Rev. B* **68**, 144514 (2003).

[39] Meier, H., Fal'ko, V.I. & Glazman, L.I. Edge effects in the magnetic interference pattern of a ballistic SNS junction. *Phys. Rev. B* **93**, 184506 (2016).

[40] Beenakker, C. W. J. Universal limit of critical-current fluctuations in mesoscopic Josephson junctions. *Phys. Rev. Lett.* **67**, 3836-3839 (1991).

[41] van Heck, B., Mi, S. & Akhmerov, A.R. Single fermion manipulation via superconducting phase differences in multiterminal Josephson junctions. *Phys. Rev. B* **90**, 155450 (2014).

[42] Groth, C. W., Wimmer, M., Akhmerov, A.R. & Waintal, X. Kwant: a software package for quantum transport. *New J. Phys.* **16**, 063065 (2014).

[43] Yao, W., Yang, S. A., & Niu, Q. Edge states in graphene: from gapped flat-band to gapless chiral modes. *Phys. Rev. Lett.* **102**, 096801 (2009).

[44] Li J., Martin I., Büttiker M. & Morpurgo A.F., Topological origin of subgap conductance in insulating bilayer graphene. *Nat. Phys.* **7**, 38-42 (2011).

[45] Zhang, F., MacDonald, A.H. & Mele, E.J. Valley Chern numbers and boundary modes in gapped bilayer graphene. *Proc. Natl Acad. Sci. USA* **110**, 10546-10551 (2013).

[46] Alden, J.S., Tsen, A.W., Huang, P.Y., Hovden, R. Brown, L., Park, J., Muller, D.A. & McEuen, P.L., Strain solitons and topological defects in bilayer graphene. *Proc. Natl Acad. Sci. USA* **110**, 11256-11260 (2013).

[47] Ju, L., Shi, Z., Nair, N., Lv, Y., Jin, C., Velasco Jr., J., Ojeda-Aristizabal, C., Bechtel, H.A., Martin, M.C., Zettl, A., Analytis, J. & Wang., F. Topological valley transport at bilayer graphene domain walls. *Nature* **520**, 650-655 (2015).

[48] Allen, M. T., Shtanko, O., Fulga, I. C., Akhmerov, A. R., Watanabe, K., Taniguchi, T., Jarillo-Herrero, P., Levitov, L. S. & Yacoby, A. Spatially resolved edge currents and guided-wave electronic states in graphene. *Nat. Phys.* **12**, 128-133 (2016).

[49] Zhu, M.J., Kretinin, A.V., Thompson, M.D., Bandurin, D.A., Hu, S., Yu, G.L., Birkbeck, J., Mishchenko, A., Vera-Marun, I. J., Watanabe, K., Taniguchi, T., Polini, M., Prance, J.R., Novoselov, K.S., Geim, A.K. & Ben Shalom, M. Edge currents shunt the insulating bulk in gapped graphene, *Nat. Commun.* **8**, 14552 (2017).

[50] Dou Z., Morikawa S., Cresti A., Wang S.-W., Smith C.G., Christos M., Kazakova O., Watanabe K., Taniguchi T., Masubuchi S., Machida T. & Connolly M.R. Imaging bulk and edge transport near the Dirac point in graphene moiré superlattices. *Nano Lett.* DOI: 10.1021/acs.nanolett.8b00228 (2018).
